# Noncommunicable Diseases Prevention Policies and Their Implementation in Africa: A Systematic Review

**DOI:** 10.3389/phrs.2021.1604310

**Published:** 2022-02-09

**Authors:** Melkamu Dugassa Kassa, Jeanne Martin Grace

**Affiliations:** ^1^ College of Health Science, Discipline of Biokinetics, Exercise and Podiatric Medicine, University of KwaZulu-Natal, Durban, South Africa; ^2^ Department of Biokinetics, Exercise, and Sports Science, Sport Academy, Jimma University, Jimma, Ethiopia

**Keywords:** health policy, NCDs, NCDs prevention and control, policy gaps, policy implementation, prevention strategies, tobacco

## Abstract

**Objectives:** To synthesize the existing evidence on NCD policy equity, policy practices, and policy implementation gaps to prevent NCDs in African countries.

**Methods:** Following the PRISMA-Extension for equity-focused review guidelines, the authors systematically searched documentary evidence from seven databases (BMC, CINHAL Plus, Cochrane, Google Scholar, PubMed, Web of Science, and Scopus) to identify studies conducted and published on African countries between April 2013 and December 31, 2020.

**Results:** From identified 213 records, 21 studies were included in the final synthesis. Major results showed inadequate studies on NCD policy, unsatisfactory NCD-related policy development, poor policy implementation, lack of policy equity to combat NCDs, and lack of data recorded on NCDs’ prevalence, morbidity, and mortality.

**Conclusion:** The rigorous WHO-endorsed NCD policies and prevention strategies on the African continent might debar African policymakers and leaders from developing and implementing indigenous NCD-combating strategies. Continent-wide innovative and indigenous NCD-prevention policies and policy equity to effectively prevent, control, and manage NCDs must be developed by African scientists and policymakers.

## Introduction

Noncommunicable diseases (NCDs) continue to be a global public health challenge faced by both developed and Low and Middle-Income Countries (LMICs), regardless of their economic and health systems [1]. In 2016, Member States of the World Health Organization (WHO) developed and implemented national action plans for NCDs in line with the Global action plan for the prevention and control of NCDs (2013–2020) [2, 3]. Globally, NCDs account for 40 million deaths annually and approximately $7.8 billion in losses [4, 5]. In association with globalization and technological advancements, the burden of NCDs increases and affects the lives, economies, and healthcare systems of both advanced and low-resourced countries [6]. Shreds of evidence show that NCDs share common risk factors such as an unhealthy diet, unhealthy lifestyles, customary alcohol use, frequent tobacco use, and physical inactivity [4, 5, 7]. Disease prevention is based on robust healthcare systems and the implementation of effective national healthcare policies. In this regard, the World Health Organization (WHO) and member nations have made many bilateral and multilateral efforts to reverse the growing burden of NCDs by setting ten goals to reduce risk factors associated with them by 2030 [1–3, 8]. To facilitate and encourage the prevention of NCDs, the WHO actioned $11 billion a year to implement a set of NCD “best buy” interventions in all LMICs, and up till now, considerable efforts have been made to design and implement policies that ensure the effectiveness of these “best buy” interventions at a national level [4, 5, 7]. If adequately and correctly implemented, the WHO’s “best buy” intervention will strategically tackle NCDs’ growing and overwhelming burden in Africa [9–11]. Despite the “best buy” interventions approved by the WHO for its member nations, most healthcare policies and disease prevention strategies in Africa focus on communicable disease prevention and management, with attention being given to the unsatisfactory prevention and management of NCDs [12, 13]. Consequently, morbidity and mortalities associated with NCDs are not timeously addressed [14], with the African continent suffering from a quadruple burden of illness comprised of infectious communicable diseases, road traffic injuries, coronavirus (COVID-19), and the growing burden of NCDs [15]. In Africa, the brain drain of healthcare workers and the current 1:5000 healthcare worker to patient ratio make the prevention of NCDs and policy implementation challenging [16]. Additionally, regarding the scarcity of healthcare workers, the current healthcare system is fragile because of maladministration, corruption, and low salaries of healthcare professionals [16].

Moreover, NCDs’ policy equity in Africa is inadequately addressed through policies and research, making healthcare systems in Africa inferior to those elsewhere [17–19]. Therefore, an adequately developed NCD prevention strategy that ensures policy equity for NCDs can conceptually influence a reduction in the time and financial cost spent on their prevention and management, consequently saving many vulnerable lives. However, little is known about the health system’s response to the prevention of NCDs, policy practices, and NCDs policy equity in African countries, where such policies may have a meaningful impact on NCDs. Well-organized and systematized NCD prevention policies and NCD policy equity are important to ensure appropriate healthcare decisions are made at the national and global level that will assist policymakers and managers in prioritizing healthcare services and allocating material and human resources to the most impoverished community groups [17, 19].

NCDs policy equity is often defined as the absence of systematic disparities in areas of NCDs policy coverage between more and less advantaged social groups. The systematic disparities associated with circumstances that place some groups at a further disadvantage in achieving health or having opportunities to be healthy are referred to as health inequities. The success or failure of any NCDs policy initiative may be measured in terms of health equity gaps between the worst off and the rest of a given population and throughout the health gradient. Research on health equity across the globe and the link to poor health outcomes are prevalent in the scholarly literature [17, 19, 20].

Evidence shows that African countries have applied a robust policy response to noncommunicable diseases; nevertheless, vital inequalities in health services for NCDs even now alive in attaining their country population health coverage. In the face of a breath-taking increase in NCDs, policy equity coverage, public policy research, and proven authority concern in advocating equity in NCDs policies, health inequities are mounting between various populations, and there is modest testimony that NCDs policy equity coverage are being developed and implemented. Moreover, these issues typically fail to reach governments’ policy agendas, which is a critical step towards serious debate and the identification of policy options. Equity should be contained within continent-wide health coverage, yet evolving testimony proves that without satisfactory attention to recognizing NCDs policy equity coverage, vulnerable community groups may persist in getting deficient or substandard health care. Despite this increasing amount of testimony and the overabundance of suggestions by various professionals advocating for governments to adopt policies that address long-lasting inequities, exceedingly modest achievements have been made in developing and implementing NCDs policy equity coverage [17, 19–21].

Therefore, this systematic review aims to identify the existing NCD policy practices, NCDs policy equity coverage, and policy implementation gaps to prevent NCDs in African countries. Additionally, it intends to reveal the best NCD policy practices and policy equity regarding the prevention of NCDs in Africa.

## Methods

Studies conducted between April 1, 2013 and December 31, 2020 were systematically reviewed following the PRISMA-Extension for equity-focused reviews (PRISMA-E) guidelines of all existing articles published in English, including qualitative, mixed-method, and document analysis covered NCD policy practices, policy equity, and policy implementation gaps in African countries. In this systematic review, studies are constrained to African countries, including North African countries. The rationale is that, while most high-resource countries have endorsed WHO noncommunicable disease (NCD) “best buy” policies to prevent NCDs, little is known in African countries about the equity of NCD policies, their implementation gaps, and the factors affecting NCD policy implementation in Africa. While there is an urgent need for policymaking that prioritizes NCDs policy equity, successful strategies for advancing such an agenda across multiple policy sectors are not well known. This study aims to address this gap by identifying best policy practices and existing gaps in policy development and implementation to advance the policy agenda of NCDs across multiple policy domains.

### Search Strategy

The authors systematically searched documentary evidence from seven electronic databases (BMC, CINHAL Plus, Cochrane, Google Scholar, PubMed, Web of Science, and Scopus) to identify peer-reviewed articles published between April 2013 and December 31, 2020. Research conducted at global, continental, regional, national, and subnational policy levels was included to capture heterogeneous information on best policy practices, NCDs policy equity, and policy gaps to prevent NCDs. All searches were conducted directly without using an intermediate interface such as Ovid. Table 1 shows the search terms used and the Cochrane Africa screen was applied to guarantee search results to the author’s research interest [22].

**TABLE 1:** Search terms for the systematic review. (Noncommunicable Diseases Prevention Policy Implementation, Africa, 2021).

Search terms for seven databases employed: “Noncommunicable diseases (NCDs) policy” OR “NCDs policy” OR “NCDs policy process” OR “NCDs policy response” OR “NCDs policy implementation” OR “best NCDs policy practice” OR “NCDs policy gaps” OR “NCDs policy equity” OR “NCDs policy equity implementation” OR “NCDs policy equity practice” AND Cochrane Filter

### Eligibility Criteria and Selection of Studies

The following five inclusion criteria were applied to determine if the reports warranted further investigation: 1) was the research conducted in African countries, or was data from African countries used? 2) Were NCD policy practices or NCD policy equity evaluations included? 3) Were local or nationwide NCD prevention strategies described? 4) Was at least one of the four lifestyle risk factors for NCDs (alcohol use, physical inactivity, tobacco use, and unhealthy diet) included? 5) Were the results published in English?

In this study, the best policy practice is defined as NCD prevention action plans and their implementation that involve managing and addressing at least one of the four NCDs risk factors. It also includes the screening, management, and prevention of NCD risk factors at the individual, subnational, and national levels. The term “NCDs policy equity” refers to the policy equity in the coverage, treatment and management of the major NCDs lifestyle risk factors such as harmful use of alcohol, tobacco use, unhealthy dietary practice, and physical inactivity as reflected in the national NCDs policy and healthcare services on the African continent and across its countries [23]. Health inequalities include differences in length of life; quality of life; rates of disease, disability, death; severity of disease; and access to treatment due to poorly designed policy equity [23]. Two reviewers autonomously evaluated the titles and summaries of the selected studies for inclusion in the full-text review. Only those full-text studies which both authors agreed with and met the inclusion criteria were selected for full-text review eligibility. Differences were minimized based on mutual unanimity between the two reviewers. To find eligible supplementary studies, the two reviewers searched the references lists of included studies.

### Quality Assessment

For quality assessment, the two authors utilized the Combined Health Policy Evaluation reporting guide to weighing up the included studies [24, 25]. The authors performed an inclusive recording of the included studies to ensure data quality, giving a point to each satisfying detail on the specification, and excluded poor-quality studies.

### Data Extraction and Analysis

Relevant information such as country, year of publication, study method, design, study perspective, results, and conclusions was extracted from the included studies. After that, the relevant information was summarised and recorded in tables.

## Results

**FIGURE 1:**
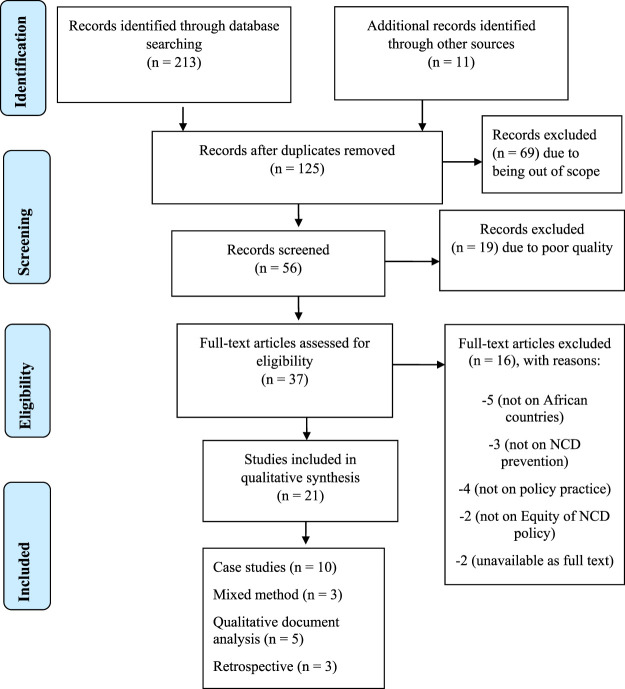
Flowchart showing the selection of studies for the systematic review. (Noncommunicable Diseases Prevention Policy Implementation, Africa, 2021).

Initially, 213 studies were identified using the search criteria depicted in Figure 1. Mendeley Desktop reference management was used to identify duplication and data management. After removing duplications, checking the eligibility of full-text articles, applying exclusion criteria, and screening, 21 papers met the inclusion criteria [10, 12, 13, 26–43].

### Study Participants and Characteristics

The included studies involved 1,163 participants, comprised of healthcare workers, key informants in the ministry of health, stakeholders, and policymakers. As displayed in Figure 1, among the included studies, the highest proportion was made up of ten case studies (47.6%), three mixed methods studies (15.8%), three studies were retrospective (15.8%), and the remaining four studies (21%) were qualitative document analysis. For information dissemination, the included studies are presented in the first African NCDs research conference in 2017, released online, posted in WHO NCDs repository, and added to different scientific databases such as google scholar, PubMed, Scopus, and Web of Science.

The overall characteristics of the studies included in this systematic review are shown in Table 2. Of the 21 studies, only one study considered health information and the prevalence of NCDs, morbidity, and mortality data for the prevention of NCDs [26]. Four studies focused on Africa’s NCD policy development process [10, 27–29], whereas the other five considered tobacco control policy effectiveness [30–33, 38]. One study considered policy response to NCDs in five African countries [34]. A mere three studies measured policy implementation on the four major NCD risk factors such as tobacco use, harmful alcohol use, unhealthy dietary practice, and physical inactivity [35, 36, 39]. Three studies considered opportunities and challenges to implementing NCD policy to combat the developing burden of NCDs [12, 37, 40]. Most of the studies considered WHO-endorsed policy strategies branded as “best buys” and a global action plan to prevent NCDs 2013–2020 in their NCD policy analysis. Only one study focused on Africa as a whole [13], while two studies considered multi-sectoral involvement in developing and implementing NCD policy in Africa [41, 43]. The remaining study considered the monitoring of NCD policies progress; however, it did not sufficiently describe the use and inclusion of health equity in healthcare policy as a strategy to prevent NCDs in Africa [42]. The demonstrated best policy practice to combat the growing burden of NCDs was on tobacco control strategies, while physical inactivity policies were the most poorly considered policy practice for preventing NCDs in Africa.

**TABLE 2:** Characteristics of included studies (n = 21 articles, with 1,163 participants). (Noncommunicable Diseases Prevention Policy Implementation, Africa, 2021).

Authors	Setting	Study design	Sample size used	Study aims
Wisdom et al. 2018 [10]	Sub-Saharan Africa (SSA)	A multi-country policy review using a case study design	202 key informants from six countries	To describe the timelines, context, key actors, and strategies in developing and implementing the treaty and describes how six sub-Saharan countries responded to its call for action on tobacco control
Witter et al. 2020 [12]	Sierra Leone	Case study	An in-depth interview with 28 key informants and review of documents for secondary data	To explore opportunities and challenges and highlight lessons for Sierra Leone and other fragile states in the battle against the growing NCD epidemic
Nyaaba et al. 2017 [13]	Africa	Document analysis	The WHO 2011, 2014, and 2015 NCDs reports	To assess Africa’s progress towards WHO policy recommendations for reducing the NCD burden
Kassa and Grace 2019 [26]	Ethiopia	Mixed method sequential explanatory design	312 healthcare workers	To evaluate the availability and status of NCD data within the healthcare system
Mukanu et al. 2017 [27]	Zambia	Qualitative approach	In-depth interview with eight key informants	To evaluate the policy response to NCDs by the ministry of health in Zambia
Ndinda et al. 2018 [28]	South Africa	Case study	An in-depth interview with 44 key informants (2014–2016) from the health and non-health sectors	To identify the political and ideological factors that influenced the design of NCD policies
Juma et al. 2018 [29]	Five SSA countries	Retrospective case study	202 key informants from all five countries	To evaluate the NCD prevention policy development process in five African countries (Kenya, South Africa, Cameroon, Nigeria, and Malawi)
Oladepo et al. 2018 [30]	Nigeria	Case study: Walt and Gilson Policy Analysis Framework	Key informant interviews with 44 stakeholders in the public and private sectors	To examine tobacco control policies in Nigeria, the use of multi-sectoral action in their formulation, and the extent to which they align with the WHO “best buy” interventions
Mohamed et al. 2018 [31]	Kenya	Case study	In-depth interviews with 39 stakeholders such as government, civil society, and non-governmental organizations	To identify the tobacco control policy formulation and implementation as well as the associated facilitators and barriers
Mapa-Tassou et al. 2018 [32]	Cameroon	Case study	Interviews with 38 key stakeholders and field observations	To examine tobacco prevention policies in Cameroon aligned with the WHO tobacco “best buy” interventions and the level of implementation of these policies
Sanni et al. 2018 [33]	South Africa and Togo	Two-country case study	An in-depth interview with 56 key informants and document analysis on tobacco control policies	To assess the use of a multi-sectoral approach (MSA) in developing and implementing tobacco control policies in South Africa and Togo
Juma et al. 2017 [34]	Kenya	Retrospective case study design	An in-depth interview with 39 key informants	To examine policies addressing the WHO “best buy” interventions for NCD prevention
Ndinda et al. 2017 [35]	South Africa	Case study analyzing existing policies that addressed major NCD risk factors	An in-depth interview with 44 key informants	To assess the state of implementation of NCD “best buy” interventions; identify barriers to and facilitators of the formulation and implementation of NCD prevention and control policies in South Africa
Mapa-Tassou et al. 2017 [36]	Cameroon	Case study design	Review of all national policy documents, field observations, 43 in-depth interviews with policymakers and implementers	To describe the development of policies in multiple sectors designed to address NCD prevention’ best buys’ in Cameroon
Fassil et al. 2019 [37]	Ethiopia	Mixed method, triangulation design conducted in two stages	Document analysis	To examine the policy and strategy gaps in reducing the modifiable NCD behavioural risk factors in Ethiopia to inform and guide policymakers and other stakeholders
Teshome et al. 2020 [38]	Ethiopia	Retrospective review	Four databases (PubMed, Scopus, Web of Science, and Embase)	To analyze tobacco-related policies in Ethiopia that are relevant to control tobacco use and mitigate its impact
Matanje-Mwagomba et al. 2017 [39]	Malawi	Qualitative case study design	32 key informants’ in-depth interviews	To describe the extent of inclusion of alcohol-related “best buy” interventions in national policies and the application of multi-sectoral action in developing Malawi’s alcohol policies
Musango et al. 2020 [40]	Mauritius	Qualitative research design	N/A	To analyze and score the common health system challenges that impede the delivery of core NCD interventions and services in Mauritius and provide policy recommendations to address health system barriers in delivering NCD interventions and services
Ngwira et al. 2017 [41]	Malawi	Mixed methods design	In-depth interview with 32 key informants	To generate evidence on the extent to which Multi-Sectoral Action (MSA) plays a role in formulating and implementing policies related to NCD preventive “best buy” interventions in reducing risk factors for NCDs
Lamri et al. 2014 [42]	Algeria	Literature and Documents analysis	PubMed, Web of Science, Scopus, Google Scholar and Google	To understand the health policy strategy adopted by Algeria to respond to the disease
Wickramasinghe et al. 2018 [43]	Morocco and Sudan	Qualitative Case study design	Structured interviews 12 key informants	To draw on the experiences of the two countries that had made good progress in developing these MAPs in identifying best practices and barriers in their development

### Synthesis

The current review is a review of studies on NCD policy content, policy practice, policy equity, and policy gaps in African countries, and the key results of all the incorporated studies are synthesized in Table 3, under five themes, namely 1) NCD policy development process, 2) NCD policy content, 3) the policy response to NCD prevention, 4) implementation of NCD policies and 5) policy equity in NCD prevention. The five themes were discussed concurrently with the two issues to establish the best NCD policy practice and identify NCD policy gaps. The major findings show an inadequate number and scope of studies on NCD policy in African countries, indicating deficient NCD policy equity and poor implementation of existing NCD policies at subnational, national, regional, and continental levels.

**TABLE 3:** Best Noncommunicable Diseases policy practice, health equity and existing gaps. (Noncommunicable Diseases Prevention Policy Implementation, Africa, 2021).

Themes	Best NCDs policy practices	Existing NCDs policy gaps
NCD policy development process [9, 10, 13, 29]	-Enhanced global activism and governmental pledge	-The implementation of NCD policies and programs is challenging because of the underdeveloped social, economic, and political context in African countries
	-Most of the policies on NCD prevention and their risk factors comply with global guidelines such as the Framework Convention on Tobacco Control (FCTC) and the United Nations Political Declaration on the Prevention and Control of NCDs	-It focuses on a single policy such as tobacco and or alcohol
	-The involvement of important actors in tobacco policy development includes the National Department of Health, Finance, Education, Communication, and Social Affairs	-Lack of structured organization and collaboration of multi-sectoral actors for the effective development and implementation of alcohol policies
		-Inadequate allocation of sustainable finance for NCD policy development and implementation
		-An insufficient effort to mitigate the influence of the alcohol industry among multi-sectoral actors
		Harmonization challenges
		-The lack of clear, coherent outlines to guide working with other sectors
		-Great complexities in sector operations and high staff turnover make it hard to have the same individuals participating consistently and maintaining similar views
		-In many instances, people at different meetings and workshops represent some sectors. Hence, coordinating many different people along with resource issues were the most prominent challenges. Because sectors had contrasting views, synthesizing those diverse views into a single coherent plan was challenging
NCDs policy content [13, 27, 29–33]	Most of the established policies target tobacco control, with a few policies focusing on alcohol control	Policies targeting an unhealthy diet and physical inactivity are the most neglected aspects of NCD prevention strategies in most African countries
Policy response to NCD prevention [10, 12, 26, 27, 29, 41, 43]	-Health sector development of NCD prevention strategic action plans evident in almost all countries	-The poor coverage of population-based NCD intervention because of insufficient intersectoral collaboration, lack of priority setting, poor transformation supervision, inadequate human resources, poor community liberation, and limited political pledge
	-Generating evidence through steps survey and setting time-bound national targets on NCD behavioural risk factors available in some countries	-Limited integration of evidence into practice; inadequate application of information and technology solutions
	-Confronting tobacco use and smoking by banning their promotion, increased taxation, and displaying posters that reflect tobacco’s effects on health are prominent in Kenya, Malawi, Nigeria, Cameroon and South Africa	Antagonism between administration divisions
	-The taxation of sugar-sweetened beverages (SSBs) to prevent and control NCDs related to unhealthy diets in South Africa	Competition among sectors, particularly related to the leadership of some policies. For instance, in Malawi, during the development of the alcohol policy, there was a conflict between the Ministry of Health and the Ministry of Trade and Industry about who would lead the process. This competition also affected implementation. In Nigeria, during the formulation of the Tobacco Act, there was a competition between the Federal Ministry of Health and the regulatory organizations over who was the most appropriate ministry to lead the tobacco control policy. In South Africa, the Department of Trade and Industry’s priorities and those of Treasury clashed with the Department of Health and Social Development. As a result, passing a bill to ban alcohol advertising became complicated and fraught and was eventually withdrawn
	-The current high taxation rate on alcohol products in Ethiopia to reduce the use of alcohol	
Implementation of NCD policies [11, 34–36, 39, 41, 43]	-Execution of the ratified WHO Framework Convention on Tobacco Control (FCTC) addressed tobacco and alcohol control policies	Implementation gaps
	-Recent tobacco-related policies were established through strong multi-sectoral commitment and covering all four WHO “best buy” interventions	The implementation levels varied widely from one policy and country to another because of inadequate funding, limited institutional capacity, inadequate action across different sectors within and outside the health system, and a lack of standardized monitoring and evaluation mechanisms to inform policies
		The dearth of administrative motivation
		Inadequate administration will hinder both policy formulation and multi-sectoral action. In most countries, governments were slow in acting and often lacked the political will to formulate policies to address NCD risk factors. In Malawi, this is also shown by the failure to ratify the WHO FCTC. In Cameroon, the government granted massive subsidies to tobacco farmers in the country
Policy equity in NCD prevention [17, 19–21, 30–33]	-Integration of social determinants of health in all public policies, development of innovative health financing policies is implemented in Botswana	Variances in interests, urgencies and aims
	-Improved school-based deworming coverage through intersectoral coordination in Kenya to provide health education to parents and pupils in their respective schools	In South Africa, the departments of Social Development, and Health and the police are concerned about the negative health impact of alcohol use, while the departments of Finance, as well as Trade and Industry, are concerned about the loss of revenue from taxing alcohol consumers and the consequent job losses in the alcohol and advertising industries
	-The intersectoral action to reduce the key determinants of NCDs such as social and economic factors that affect the health of vulnerable groups in Swaziland	Deficiency of sufficient assets
		-Most of the countries reported insufficient financial resources allocated to develop NCD prevention policies and engage multiple sectors in policy implementation activities. Inadequate finances and human resource capacity meant that policies were not implemented. There is an over-reliance on NGOs to support certain aspects of health equity policy formulation and implementation
		Deficiency of mindfulness by the important subdivisions
		There was a lack of awareness about NCDs and their risk factors amongst the populations in most countries. In countries such as Kenya, Cameroon, Malawi, and Nigeria, NCDs had not been given priority in the past as compared to communicable diseases, and awareness among non-health sectors was even lower. Many sectors, other than the health sector, were unaware of their potential contributions to NCD prevention. NCD prevention was assumed to be a health sector issue that must spearhead policy development to address these risk factors

## Discussion

### NCD Policy Development Process

This systematic review exemplifies a substantial number of NCDs policy practices, challenges, and gaps among African countries—as shown by the nationwide position evaluations—and the NCDs policy strategies that nations implement to prevent the growing burden of NCDs in Africa. The current study’s results concur with findings by other researchers that the implementation of NCD policies and programs is challenging because of the underdeveloped social, economic, and political context in African countries with a need for enhanced global activism and governmental pledge [44]. Substantiated by other researchers, most policies focus on a single policy, such as tobacco or alcohol [45], with an insufficient effort to mitigate the alcohol industry’s influence among multi-sectoral actors to develop and implement alcohol policies effectively. Conversely, policies on NCD prevention and their risk factors comply with global guidelines such as the Framework Convention on Tobacco Control (FCTC) and the United Nations Political Declaration on the Prevention and Control of NCDs [46]. In addition, there is a lack of clear, coherent outlines to guide working with other sectors such as trade and industry [13, 29]. However, the involvement of important actors in the development of the tobacco policy, such as the National Department of Health, Finance, Education, Communication, and Social Affairs, is noted, which is substantiated by other researchers [10, 13]. Significant complexities in sector operations and high staff turnover are evident, making it hard to have the same individuals participating consistently and maintaining similar views [30–33]. In agreement with other findings, there is a lack of structured organization and collaboration of multi-sectoral actors [47, 48]. In many instances, people at different meetings and workshops represent some sectors like the health sector without sectors like trade, industry, and revenue. Hence, coordinating many different people and resource issues were identified as prominent challenges. Also, because sectors had contrasting views, it was challenging to synthesise those diverse views into a single coherent plan. The NCD policy development process is probably the biggest challenge because of the inadequate allocation of sustainable finance for NCD policy development and implementation [10, 13, 27, 29].

### NCDs Policy Content

Most of the established policies target tobacco control, with a few policies focusing on alcohol control. However, policies targeting an unhealthy diet and physical inactivity are the most neglected NCD prevention strategies in most African countries [44, 45].

### The Policy Response to NCD Prevention

Although health sector development of NCD prevention strategic action plans is evident in almost all countries, there is inadequate coverage of population-based NCD interventions because of insufficient intersectoral collaboration, lack of priority setting, poor transformation supervision, inadequate human resources, poor community liberation, and limited political pledge. Consistent with other studies [49], there is evidence of generating policies in some countries responding to NCD prevention through steps surveys and setting time-bound national targets on NCD behavioural risk factors. For example, in Kenya, Malawi, Nigeria, Cameroon and South Africa, the promotion of tobacco use and smoking were banned, taxes were increased, and displaying posters that reflect tobacco’s effects on health were displayed [29]. Also, in South Africa, the taxation of sugar-sweetened beverages (SSBs) to prevent and control NCDs related to unhealthy diets and the current high taxation rate on alcohol products in Ethiopia to reduce alcohol use are NCD policy response examples [35]. However, contradictory to the above, the current research confirms other researchers’ findings identifying limited integration of evidence into practice; inadequate application of information and technology solutions in some cases due to competition amongst industry sectors that are mainly related to the leadership of some policies. For instance, in Malawi, during the development of the alcohol policy, there was a conflict between the Ministry of Health and the Ministry of Trade and Industry about who would lead the process [41], consequently affecting the implementation of the policy. In Nigeria, during the formulation of the Tobacco Act, there was a competition between the Federal Ministry of Health and the regulatory organizations over who was the most relevant ministry to lead the tobacco control policy. In South Africa, the Department of Trade and Industry’s priorities and those of Treasury clashed with the Department of Health and Social Development. As a result, passing a bill to ban alcohol advertising became complicated and fraught and was eventually withdrawn [29].

### Implementation of NCD Policies

The ratified WHO Framework Convention on Tobacco Control (FCTC) addressed tobacco and alcohol control policies. The recent tobacco-related policies established through strong multi-sectoral commitment and covering all four WHO “best buy” interventions show examples of NCD policies implementation. The multistage delay of tobacco policy adoption is principally due to political structures and policy hierarchy, complex bureaucracy, unclear roles and responsibilities, and a high degree of corruption [46]. Researchers corroborate the author’s findings on the NCD policies implementation gaps in that the implementation levels varied widely from one policy and country to another because of inadequate funding, limited institutional capacity, inadequate action across different sectors within and outside the health system, and a lack of standardized monitoring and evaluation mechanisms to inform policies [50]. Inadequate administration also hinders policy formulation and multi-sectoral action, and in most countries, governments were slow in acting and often lacked the political will to formulate policies to address NCD risk factors [51, 52]. Malawi exemplifies this by failing to ratify the WHO FCTC [41], whereas, in Cameroon, the government granted massive subsidies to tobacco farmers in the country [32].

### Tobacco Control Policies

A couple of African countries such as Nigeria and South Africa had policies since the 1990s on the main NCDs risk factors, far earlier than the global ambition to combat NCDs [38, 46]. In Nigeria, a national tobacco control policy dates back to the 1950s. However, the Federal Ministry of Health developed the current tobacco-related policies through strong multi-sectoral engagement and covering all the four WHO “best buy” interventions [38]. In Kenya, the comprehensive tobacco policy was developed in 2007 with five chief enablers to the policy formulation and implementation, including political commitment and strong leadership, a coordinated mechanism, stakeholder passion and commitment, resources, and the constitutional requirement for inclusion of stakeholders [37].

In Cameroon, 12 of 19 tobacco use and prevention policies address the WHO “best buy” interventions. The good news is that Cameroon’s policy formulation was driven locally by the social context of noncommunicable diseases and globally by the adoption of the WHO Framework Convention on Tobacco Control. These policies incorporated all four domains of the tobacco use “best buy” interventions to some extent. Formulating policies on smoke-free areas was single-sector-oriented while determining tobacco taxes and health warnings was more complex, utilizing multi-sector approaches [11, 35, 46]. The study in six African countries such as Cameroon, Kenya, Nigeria, Malawi, South Africa, and Togo revealed that multiple stakeholders, including academics and activists, led a rigorous effort to push the WHO treaty forward on tobacco despite counter-marketing from the tobacco industry. However, the six countries responded uniquely in applying their tobacco policies, with variances linked to the country’s socio-economic context, primacies of country leaders, industry existence, and choice of strategies [11, 35].

The results of the current review in two African countries reveal that the stakeholders involved in South Africa were more diverse, proactive, and dynamic than those in Togo; comparatively, the strategies employed in Togo were more straightforward. The extent of understanding and use of a multi-sectoral approach in both countries consisted of an inter-sectoral action for health, whereby the health department struggled to collaborate with other sectors within and outside the government [40]. A study in Ethiopia indicates that the country has ratified and is underway to implement tobacco control policies and strategies [39]; however, its application is challenging, with tobacco consumption currently ever-increasing [39].

### Alcohol Control Policies

The findings of the current review reveal that the development and implementation of alcohol policies are not satisfactory. A study in Malawi shows that three of the 12 national alcohol policy documents considered at least one “best buy” intervention [43]. The results further show that alcohol policy processes have been slow in Nigeria and Malawi [44]. Effective development and implementation of alcohol policies require structured organization and collaboration of multi-sectoral actors. Sustainable financing mechanisms for the policy development and implementation processes should be considered, and the alcohol industry’s influence should be mitigated.

### Unhealthy Diet Control Policies

An unhealthy diet is one of the major lifestyle risk factors leading to NCDs; hence it requires a strong policy response. However, the salt policy is the only available indicator employed by the WHO to evaluate the improvement of an unhealthy diet [45]. The WHO endorsed salt policies include four “best buys” such as reconvening and setting a goal of salt in diets, encouraging a supportive atmosphere for lower sodium options, endorsing behaviour alteration using media campaigns, and applying packet tagging [14, 45]. Among African countries, South Africa implemented nationwide salt and sodium decrease programs aiming at packet tagging and product reformulation in the form of a voluntary salt decline in processed diet and snacks [35]. A guideline in South Africa for the obligatory platform tagging of salt, fat, sugar-sweetened beverages, and energy intake using the guided daily amount, was presented but not implemented yet [35, 46]. Cameroon, Kenya, Malawi, and Nigeria have drafted salt reduction strategies; however, an explicit policy on salt decrease was not yet available [32–34, 36, 44]. Population behaviour change strategies, such as creating awareness on high salt intake and empowering people to change their behaviours have been introduced in Kenya and Malawi [32, 34].

In Cameroon, posters were displayed to raise awareness about the reduction of salt intake. Mass media was used for community consciousness on reduced salt intake in food and the replacement of trans fat with polyunsaturated fat in diets. However, there were no clear policies concerning the WHO “best buys” [33]. The application of MSA in the implementation of salt reduction policies require creating awareness regarding the benefits of reduced salt intake so that the public can begin to demand their rights when making purchases of processed foods in retail outlets or restaurants, with monitoring of processed food to ensure compliance [32–34]. Although applying a multi-sectoral approach is ideal in policy formulation, the approach does not guarantee inclusion and participation of all critical stakeholders, as participation is voluntary and without incentives [32–34].

### Physical Inactivity Policies

Existing evidence shows that implementing public education and awareness campaigns is the best method to promote physical activity [4, 14, 45]. Up-to-date, most African countries have not implemented any programs that support behavioural change regarding physical inactivity except South Africa. The Global action plan on physical activity (2018–2030), adopted by the World Health Assembly resolution (WHA71.6), urged the WHO member states to implement the promotion of physical activity to develop global monitoring and reporting systems [53]. However, policies and programs targeting physical activity have yet to yield tangible results in Africa [32–34, 36].

In Cameroon, community consciousness was raised using mass media on the importance of physical activity to maintain health and fight NCDs [33]. In terms of physical activity, a variety of stakeholders endorsed physical activity for different motives. A multi-sectoral approach in implementing physical activity programs must take place by design, not by default. Even though physical activity is gaining some traction as a fundamental part of the public health agenda, physical activity surveillance, policy, and research in Africa are in their infancy. Even though there is no national physical activity/inactivity data in Africa, small scale specific group data shows that some countries such as Cameroon, Kenya, Malawi, Nigeria, and South Africa show signs of a physical activity transition characterized by a concerning shift from high-activity lifestyles to sedentary lifestyles [32–35].

There is also a realization that if left unaddressed, physical inactivity in the coming years will cause more people to suffer several NCDs associated morbidity and mortality, and NCDs will rise [32, 33]. As physical activity is not a nationally promoted activity, many people do not engage in it. However, as some African countries economies’ steadily grow, and there are reported improvements in incomes [35, 42]; with these changes, significant shifts occur in diets and physical activity patterns. As people become financially stable, they tend to gravitate towards unhealthy habits, mainly because they can afford such a lifestyle; hence healthy diets and physical activity guidelines are needed [32–35, 42, 44, 49].

In most African countries, there is no inclusive national standalone physical activity policy; however, there is a national physical activity policy draft and a few group-specific policies in a few countries. For instance, Kenya and South Africa have shown substantial improvement concerning encouraging physical activity, public health research, and surveillance. However, there are several possibilities for progress; for instance, physical activity can be placed as urgent on the public health program and to request a multi-sectoral approach in endorsing physical activity [32, 35]. Encouragement to engage in physical activity tends to keep youth away from drugs and crime. In South Africa, the Department of Health and Education progressively establishes platforms to address physical inactivity in public institutions [35, 46]. Creation of physical activity institutions as well as spaces for community physical activity practices; introduction of physical activity programs in secondary schools and organization of physical fitness programs in both primary and secondary schools as well as universities; and transferring physical activity from the state to municipalities, as well as mass media coverage, is also recommended [33].

### African Regions and Countries With Less/Higher Impact Concerning NCDs Policy Implementation

Since 2015, most African countries are developing and implementing NCDs policies at the national level despite its poor implementations. As illustrated in Table 4, West and East Africa regions lead with the highest integrated NCDs policies developed, namely 12 (21.8%) and nine (16.3%). Tobacco policies, six (11.0%) and five physical activity policies (9.0%), are highly implemented NCDs policies in East Africa, while it is least implemented in North and Southern African regions. The implementation of dietary policies, namely six (11.0%) and 11 tobacco policies (20.05%), is highest in West Africa, followed by Central and East Africa, respectively, while these policies in North and Southern African regions are the least. In terms of overall policy implementation, tobacco policies are the highest implemented policy, namely 26 (47.3%), while nine physical activity policies (16.3%) are the less implemented NCDs policies in Africa. The five countries with the highest number of NCDs policies implementation are South Africa (10), Cabo Verde (9), and Kenya (7), Mali, Mauritius, Senegal, and Tunisia (6) [53–57]. The inadequate implementation of NCDs policies in African regions might impact other policies, particularly communicable disease policies such as HIV/AIDS, malaria, tuberculosis, maternal and under five child mortalities, affected and impacted the development and implementation of NCDs policies most African countries.

**TABLE 4:** Regions of African with the least and highest implementation of Noncommunicable Diseases policies. (Noncommunicable Diseases Prevention Policy Implementation, Africa, 2021).

Regions	Alcohol policiesN (%)	Dietary policiesN (%)	Physical policiesN (%)	Tobacco policiesN (%)	Integrated NCDs policiesN (%)
North Africa	1 (1.8)	1 (1.8)	1 (1.8)	2 (3.6)	5 (9.0)
CA	2 (3.6)	4 (7.3)	-	3 (5.5)	5 (9.0)
West Africa	2 (3.6)	6 (11.0)	2 (3.6)	11 (20.0)	12 (21.8)
East Africa	3 (5.5)	3 (5.5)	5 (9.0)	6 (11.0)	9 (16.3)
SA	2 (3.6)	3 (5.5)	1 (1.8)	4 (7.3)	7 (12.7)
Total	10 (18.1)	16 (29.0)	9 (16.3)	26 (47.3)	38 (69.09)

Note: CA, Central Africa; SA, Southern Africa.

### NCD Policy Implementation Gaps and Challenges

The authors’ findings illustrate various barriers to bringing sectors together to develop policies that address the increasing burden of NCDs in the African region. In most African countries, the establishment and application of NCD prevention policies encounter different complex challenges and gaps. For instance, there is a lack of NCD prevention situation reports, political discord, and inadequate inclusive and cohesive NCDs risk factor prevention policies. Added to these challenges are the absence of a platform for combating and managing NCDs at a national and continental level, no obedience to platform formulation standards, inadequate NCD prevention in the healthcare system, and an under prioritization of NCDs truncated assets allocation [30–33]. Moreover, the author’s finding indicates poor access to information on the morbidity, mortality, and prevalence of risk factors related to NCDs in Africa because of a lack of standardized protocols to record NCD data and poor periodic evaluation of NCD data [26]. Moreover, there is a lack of knowledge on NCDs, shortages of medication, and shortages of nurses in the clinics, which results in patients having to wait in long queues at clinics, the lack of training of healthcare providers on the management of NCDs, and the lack of supervision by the health department managers, with management guidelines for NCDs being poorly disseminated [15, 16]. The main barriers were multi sectors inadequate awareness of their potential contribution towards healthcare, inadequate commitment, conflict of interest in the case of tobacco and alcohol policies, low political will, coordination complexities, and inadequate resources [33, 40], which makes implementations of NCD policies and programs challenging [12, 13, 43]. Physical activity policies are not well developed, resulting in a lack of effective physical activity policies [13, 33, 58].

The dearth of NCD policies is attributable to the political climate in post-colonial Africa that set a diverse path for the states that were directed to tackle deep-rooted inequality [30–36]. Also, the lack of studies on specific and overarching NCD policies has slowed and fragmented the implementation of existing strategies to prevent and control NCDs and their determinants. Africa’s health systems are not prepared to deal with the rising burden of NCDs, although there are random initiatives to improve this situation [13, 42]. Also, in some African countries, the few established policies and strategies are not implemented due to poor law enforcement and administrative will [30–33]. Moreover, there is limited government and donor commitment to finance the implementation of national NCD policies and strategies. Added to these factors are limited and poorly distributed health workforce and pharmaceuticals, high financial barriers for users, and lack of access to quality-assured medicines with a resultant high alternative to private and informal healthcare quest [13]. Also, the lack of funding and conflict of interests to protect citizens from tobacco’s harmful effects versus the industry’s economic gains are the major barriers that slowed policy processes [34–38]. The main barriers to policy formulation and implementation were industry interference, lack of resources, poor enforcement, and clear roles [37]. Insufficient resource allocation and barriers associated with poor collaboration among multiple sectors [33, 39], financial constraints, high personnel turnover in different government departments, role confusion between sectors and some interference from the alcohol industry further exacerbate the problem [34, 40].

Another notable finding from the current systematic review is that most studies considered a single policy implementation to revert the growing burden of NCDs rather than developing and implementing an all-inclusive policy [59, 60]. This might be because the rigorous WHO-endorsed NCD policies and prevention strategies on the African continent debarred African policymakers and leaders from developing and implementing indigenous strategies for combatting NCDs. Therefore, African scientists and policymakers must establish continent-wide ground-breaking and indigenous NCD prevention policies and policy equity to effectively prevent, control, and manage NCDs. A comprehensive NCDs prevention policy developed and implemented with a collective set of resources has a more significant impact on efficiently preventing and controlling the burden of different NCD risk factors than a single-based health policy in terms of scope, cost, and time.

### Policy Equity in NCD Prevention

The results confirm the evidence of policy equity in NCD prevention. For example, in Botswana, there is an integration of social determinants of health in all public policies and the development of innovative health financing policies; and in Kenya, through an intersectoral coordinated school-based deworming program to provide health education to parents and pupils in their respective schools [19–21]. In Eswatini, for example, it is apparent in an intersectoral action to reduce the key determinants of NCDs, such as social and economic factors that affect the health of vulnerable groups [20]. However, contrary to the above evidence, there are, unfortunately, variances in interests, urgencies, and aims regarding policy equity in NCD prevention. For example, in South Africa, the departments of Social Development, and Health and the police are concerned about the negative health impact of alcohol use, while the departments of Finance, as well as Trade and Industry, are concerned about the loss of revenue from taxing alcohol consumers and the consequent job losses in the alcohol and advertising industries [21]. Most countries reported insufficient financial resources to develop NCD prevention policies and engage multiple sectors in policy implementation activities. Hence, inadequate finances and human resource capacity meant that policies were not implemented. Regrettably, in agreement with other researchers, NGOs are over-reliant to support specific aspects of health equity policy formulation and implementation [61]. A final gap identified is the deficiency of mindfulness by the important subdivision with a lack of awareness about NCDs and their risk factors among most countries. For instance, in countries such as Kenya, Cameroon, Malawi, and Nigeria, NCDs had not been given priority in the past as compared to communicable diseases, and awareness among non-health sectors was even lower [43, 62]. Compared to the health sector, sectors like trade and industry were unaware of their potential contributions to NCD prevention; NCD prevention was assumed to be a health sector issue that must spearhead policy development to address these risk factors [43, 59, 62]. Supporting the overall finding of the current study, a geopolitical analysis of 151 countries on NCDs policies implementation reveals that low resourced countries in Africa ranked bottom 20 by the cumulative implementation of NCDs policies [44], contrasting high resourced European and western countries where the majority of established NCDs policies are implemented [44, 63–66]. This might result from a lack of good governance and political commitment, scarce well-trained human capital to design and implement strong NCDs policies, high rates of healthcare workers brain drain, and mounting tolls of premature NCDs mortality in Africa compared to other world regions.

### Conclusion

The systematic review reveals critical gaps and extensive challenges in NCD policy processes rather than achievements in African countries, although some progress has been made since implementing their national action plans on NCDs control. The review demonstrates the sparsity of evidence regarding the implementation of NCD policy in African countries. Africa’s NCDs policy equity coverage and NCDs policy responses remain poor for reasons that are not revealed. However, the possible justification may be that African countries’ NCDs policy implementation may have several interconnected challenges such as poor awareness, inadequate inter-sectoral coordination, and funding that are national-level challenges. There is difficulty in translating policy strategies with the integration of these strategies influenced at the operationalization level. Adaptive measures surrounding funding, coordination and capacity building are crucially needed.

Accordingly, policymakers and regulatory bodies need to strengthen the inclusion and implementation of “best buy” interventions into legislation and policies related to NCD prevention across all sectors. Improved harmonization, partnerships, and sustainable joint-financing mechanisms are indispensable for the implementation of NCDs prevention. To solve implementation challenges, African health departments, the WHO, and other global allies must build up reliable monitoring strategies on harmful alcohol use, tobacco use, unhealthy dietary practice, physical inactivity, and create a health system that can ensure these policies are implemented and actioned on. The socio-cultural and economic context in the continent and the mere focus on infectious diseases prevention and control impacted and significantly influenced the implementation of established NCD policies that can prevent NCDs in Africa. Hence, Africa’s regulatory bodies must understand NCD policy practices and the extent of the intervention strategies and apply them to their unique population and environment to ensure NCDs and their risk factors are adequately addressed. Based on available evidence, the rigorous WHO-endorsed NCD policies and prevention strategies on the African continent might debar African policymakers and leaders from developing and implementing indigenous NCD-combating strategies. Continent-wide innovative and indigenous NCD-prevention policies and NCD policy equity to effectively prevent, control, and manage NCDs must be developed by African scientists and policymakers.

### Limitations

The exclusion of unpublished research and the merely English language consideration may result in publication bias and increased language partiality. Besides, the lack of comprehensive NCD policy and policy equity research and no extensive finding in all African countries may limit the generalization of the results to the context of all African countries. Furthermore, most of the studies emphasized tobacco control policies, and the findings might not be transferable to other NCDs prevention policies such as the harmful use of alcohol, unhealthy dietary practice, and physical inactivity, underlining the necessity for an auxiliary investigation into these risk factors.

## Data Availability

The original contributions presented in the study are included in the article, further inquiries can be directed to the corresponding author.

## Abbreviations

EPHI, Ethiopian Public Health Institute; FCTC, framework convention on tobacco control; FMoH, Federal Ministry of Health; LMICs, low and middle-income countries; MDGs, millennium development goals; MSA, multi-sectoral action; NCDs, noncommunicable diseases; PA, physical activity; SSA, sub-Saharan Africa; SSBs, sugar-sweetened beverages; WHO, World Health Organization.
